# Global trends in tick research: a comprehensive visualization and bibliometric study (2015–2024)

**DOI:** 10.3389/fcimb.2025.1697791

**Published:** 2025-10-28

**Authors:** Hangli Su, Maoqing Gong, Lijuan Liu, Zhaoan Sheng, Benguang Zhang

**Affiliations:** ^1^ Shandong Institute of Parasitic Diseases, Shandong First Medical University & Shandong Academy of Medical Sciences, Jining, China; ^2^ School of Public Health, Shandong First Medical University & Shandong Academy of Medical Sciences, Jinan, China; ^3^ Department of Pathogenic Biology, Jining Medical University, Jining, China

**Keywords:** tick, tick-borne diseases, tick-borne pathogens, visual analysis, bibliometric analysis

## Abstract

**Introduction:**

Ticks are ectoparasitic blood-sucking arthropods. As key disease vectors, the pathogens transmitted by ticks pose significant threats to livestock and global public health.

**Methods:**

We searched the Web of Science and Scopus databases for global literature on ticks published between 2015 and 2024. Using VOSviewer and CiteSpace software, we conducted bibliometric and visualization analyses of the national, institutional, journal, author, keyword, and reference data from the relevant literature. The aim was to assess the characteristics of global tick-related scientific research, identify research hotspots, and explore future trends in this field.

**Results:**

The study comprised 13,499 valid articles. The United States led with 30.85% of the articles, followed by China (10.46%) and Brazil (9.99%). Marcelo B. Labruna, a Brazilian author, demonstrated the highest productivity. The institution with the most articles was Universidade de São Paulo, and the journal *Ticks and Tick-borne Diseases* had the largest number of publications. Keywords related to tick-borne diseases and pathogens, such as “Lyme disease”, “tick-borne encephalitis and tick-borne encephalitis virus”, “*Borrelia burgdorferi*”, and “*Rickettsia*”, appeared relatively often, while keywords such as “One Health” and “antimicrobial resistance” have emerged in recent years.

**Discussion:**

The study of ticks and the diseases they transmit, as well as the pathogens they carry, has always been a focus for researchers worldwide. Under global climate change, the diversity of tick-borne pathogens is expanding, as evidenced by their increased geographical distribution patterns. Therefore, research is increasingly moving toward multidisciplinary and multi-sectoral approaches, aiming to safeguard the environment and to protect the health of humans and livestock through the establishment of systematic tick control systems.

## Introduction

Ticks are obligate ectoparasites that feed on the blood of a wide variety of vertebrates, including amphibians, reptiles, birds, and mammals (de La Fuente and Kocan, 2006). Ticks are vectors of numerous pathogens that cause human and animal diseases, including Lyme disease, tick-borne encephalitis (TBE), anaplasmosis, babesiosis, and others (Estrada-Peña and Jongejan, 1999; Parola and Raoult, 2001; de La Fuente and Kocan, 2006). The global dissemination of tick-borne diseases (TBDs) represents an increasing challenge to public health, given the worldwide proliferation of tick populations (Madison-Antenucci et al., 2020). The costs of these diseases are considerable, not only in terms of human health but also in losses to livestock (Jongejan and Uilenberg, 1994; Brites-Neto et al., 2015), with annual estimates ranging from $13.9 billion to $18.7 billion globally (De Clercq et al., 2012; Sungirai et al., 2018). Recent studies have shown that changes in climate, land use, and human activities have precipitated shifts in tick distributions, thereby augmenting the prevalence of TBDs (Gray et al., 2009; Estrada-Peña et al., 2012; Semenza et al., 2022). As a result, the research community has increased its focus on the ecology, transmission dynamics, and control of ticks and their pathogens (de La Fuente et al., 2023). However, despite a growing body of literature, there is a lack of systematic integration and analysis that provides a comprehensive global overview of trends, hotspots, and emerging themes in tick research.

Bibliometrics is a field of study that utilizes statistical methods to evaluate the current state of research and forecast future trends in academic scholarship (Broadus, 1987; Ninkov et al., 2022). One of the primary objectives of this discipline is to provide both quantitative and qualitative analyses of the structure, impact, and dynamics of academic communication (Ninkov et al., 2022; Hassan and Duarte, 2024). Software tools such as VOSviewer and CiteSpace enable the aggregation, processing, analysis, and visualization of data related to research publications, citations, keywords, and related concepts. These tools enable the examination of research trends, influence, and collaboration, providing valuable insights into the evolution of research subjects and facilitating the identification of emerging research domains.

The objective of this study was to conduct a global bibliometric analysis of research related to ticks published between 2015 and 2024. Using advanced tools such as VOSviewer (van Eck and Waltman, 2010, 2014) and CiteSpace (Chen, 2004, 2006), we identified pivotal research themes by mapping knowledge structures and highlighting the most significant developments in tick research. By analyzing publication trends, author collaboration networks, and the co-occurrence of keywords, this study provides valuable insights into the current state and emerging areas of tick-related research. The results will contribute to strategic decision-making in the prevention and management of TBDs, particularly in the context of a constantly evolving global landscape.

In addressing the current state of tick research, this study sought to answer the following questions:

1. What advancements have been made in tick research over the past decade?
2. What are the prevailing research trends and hotspots in tick-related studies?
3. What are the future directions for tick research, and where do the greatest opportunities for advancing knowledge in this field lie?

## Materials and methods

### Database selection

The bibliometric methodology employed in this study referred to previously published research (Öztürk et al., 2024). WoSCC is esteemed for its rigorously curated content and strong coverage of high-impact, foundational literature, making it a cornerstone for citation analysis. Conversely, Scopus offers more extensive coverage of emerging and regional journals, thereby ensuring a broader and more inclusive perspective of the scientific landscape. Thus, these databases were deemed appropriate for searching studies relevant to tick research and obtaining a comprehensive review of the extant literature in this field.

### Search strategy

The data for this study were retrieved from the Science Citation Index Expanded (SCI-Expanded) of the WoSCC and Scopus databases, covering the period from March to April 2025. A topic search was made using the keywords “tick” or “ticks.” Papers with these words in the title, abstract, author keywords, or keywords plus were mined. Articles published in English between January 1, 2015, and December 31, 2024 were included, excluding preprints or predictive data. We only selected the publication types of “Article” and “Review.” Ultimately, a total of 13,751 records from the WoSCC and 14,981 records from Scopus databases were obtained.

### Inclusion and exclusion criteria

Specific inclusion and exclusion criteria were employed in this study to ensure the selection of relevant literature. We focused on articles and reviews, particularly original articles, to ensure the inclusion of significant research findings. The inclusion criteria limited the selection to studies published in English and indexed in the Web of Science and Scopus databases. Meeting abstracts, proceedings papers, editorial material, letters, lectures, and duplicate literature were excluded.

### Quality management and data extraction

To ensure methodological rigor, this study implemented a set of stringent quality controls. The data extraction process was initiated through a systematic screening of article titles and abstracts. Subsequently, each selected publication was evaluated against the predetermined inclusion and exclusion criteria. Full bibliographic records and cited reference data were then extracted for analytical processing. A high citation index (H-index) was obtained from the WoSCC, and journal impact factors (IF) were obtained from the 2023 edition of the Journal Citation Reports (Clarivate Analytics, Philadelphia, PA, USA).

### Data analysis

**Figure 1:**
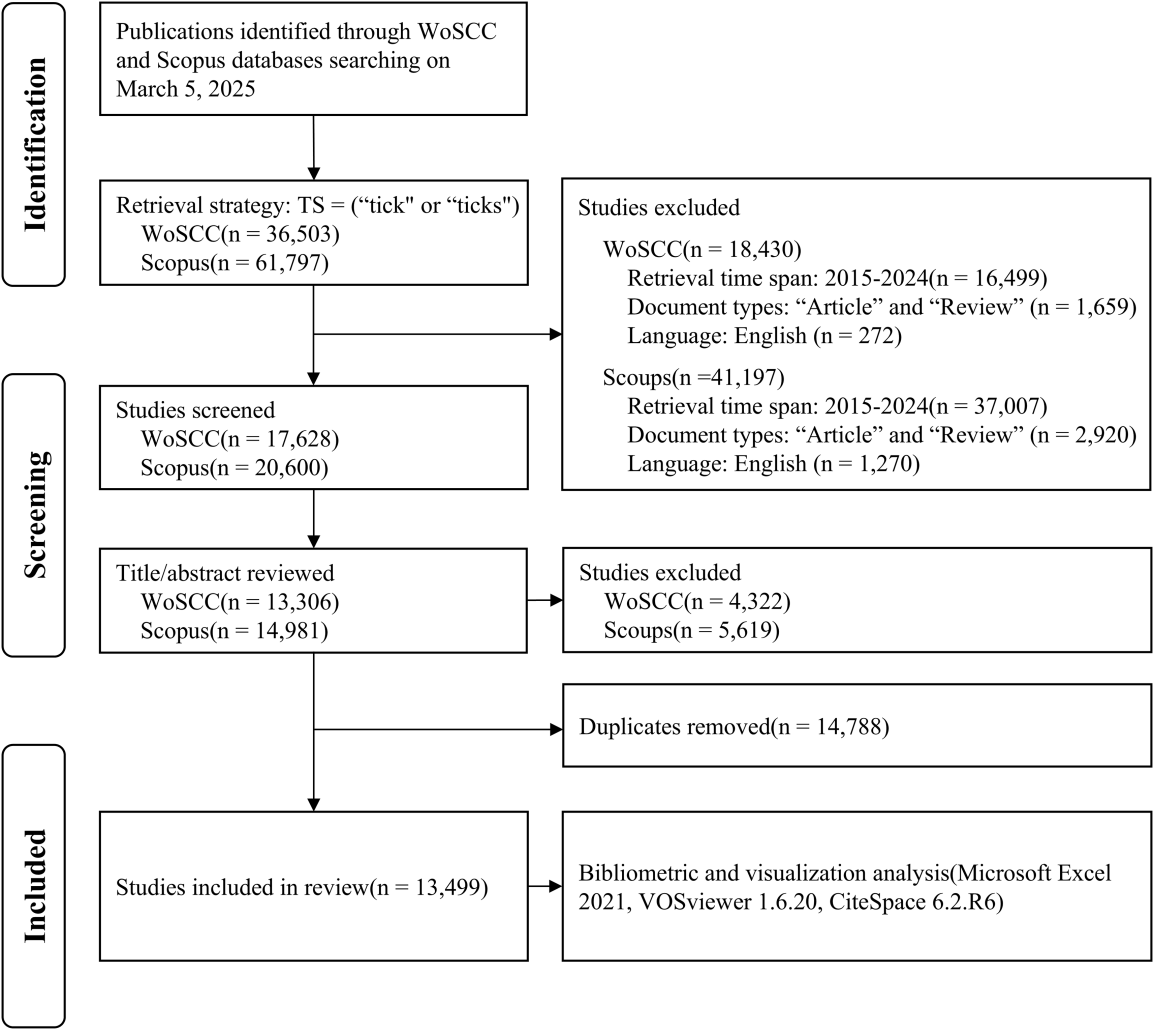
Flow diagram of the retrieval strategy and analysis. The process includes identification, screening, and inclusion phases. Initially, 36,503 publications from WoSCC and 61,797 from Scopus are identified. After excluding irrelevant studies and duplicates, 13,499 studies are included in the review. The exclusion criteria involve timeframe, document types, and language. Tools used for bibliometric and visualization analysis include Microsoft Excel 2021, VOSviewer 1.6.20, and CiteSpace 6.2.R6. WoSCC, Web of Science Core Collection.

Microsoft Excel 2021 was used to analyze the data after manual quality control. The “EndNote desktop” data format from WoSCC and the “RIS” data format from Scopus were imported into EndNote X9.1 for deduplicate deduplication. The deduplicated data were then saved as text files with the filename “download_*.txt.” Subsequently, the data were imported into VOSviewer 1.6.20 (Leiden University, Leiden, The Netherlands) and CiteSpace 6.2.R6 (Drexel University, Philadelphia, PA, USA) for bibliometric and visual analyses. VOSviewer was used to generate knowledge maps of the contributing countries/regions, institutions, influential authors and journals, co-cited references, and keyword co-occurrences. The CiteSpace application was used to extract keywords and references from the literature that exhibited the strongest citation bursts. The parameters were as follows: (1) Timespan: 2015–2024 (Slice Length = 1); (2) Selection Criteria: g-index (k = 25); (3) Pruning: Pathfinder + pruning the merged network. **Figure 1** illustrates the flow diagram of the literature search and analysis. This study did not involve human experiments and hence did not need ethical approval.

## Results

### Trends in publication and citation

**Figure 2:**
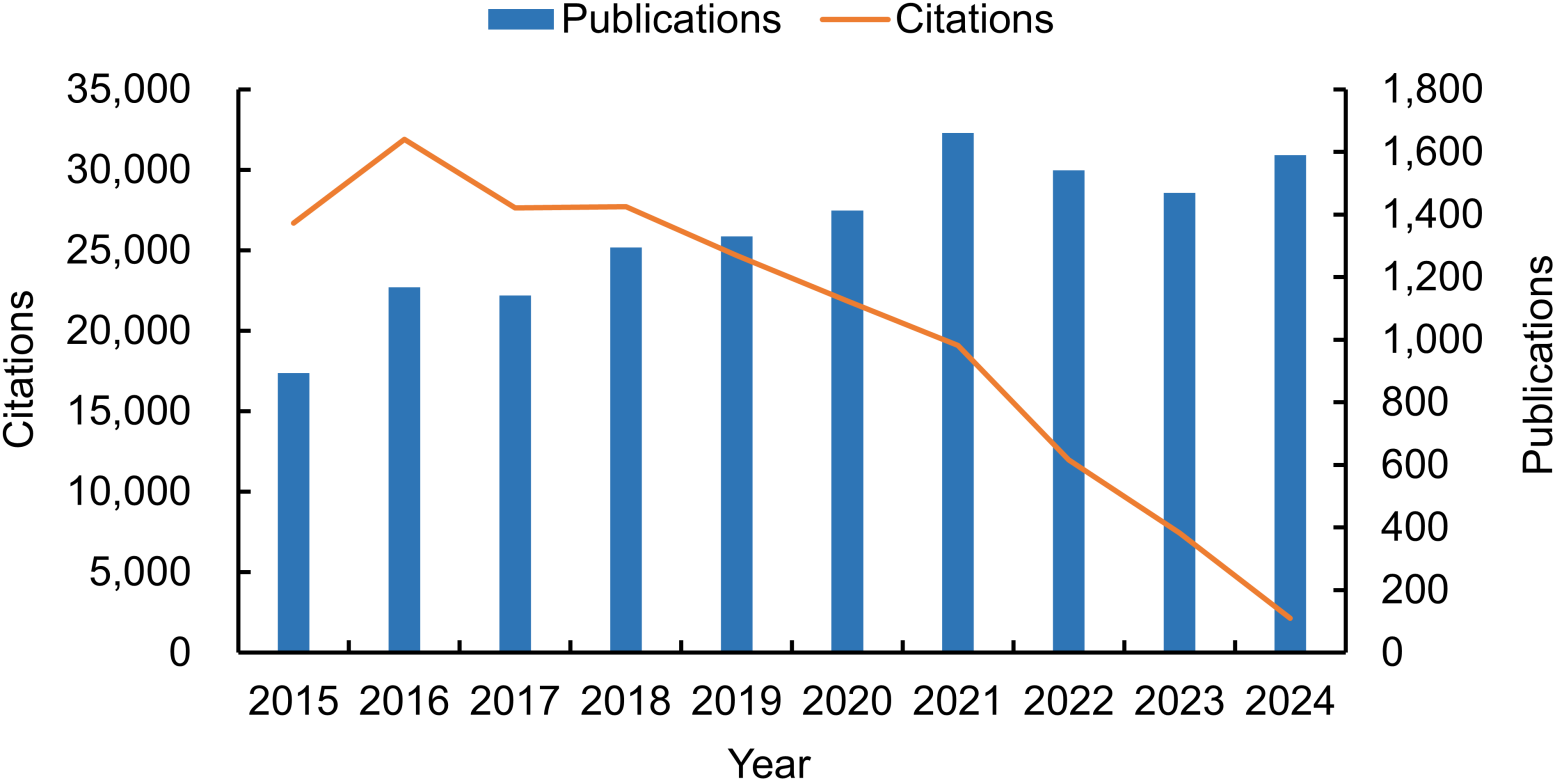
Global trends in annual publications and citations related to tick research from 2015 to 2024.

The final analysis encompassed a total of 13,499 publications, comprising 12,455 research papers and 1,044 review articles (**Table 1**). According to the search strategy, the number of annual publications steadily increased, from 893 in 2015 to 1,590 in 2024. This indicates a significant escalation in research activity, with a 78.05% increase over the 10-year period. The maximum number of papers observed was 1,660 in 2021. However, after reaching a peak in 2016, the number of citations declined year by year, with a particularly pronounced drop observed after 2020. This indicates that the pace of publication could be outpacing the citation potential of each paper. This trend could be attributed to the phenomenon of “citation lag,” where it takes several years for research to be widely cited and recognized in the academic community (Wang, 2013). As of the retrieval date, these publications had been cited 201,148 times, averaging 14.90 citations per publication (**Figure 2**).

**Table 1:** Bibliometric profile of tick research (2015–2024).

Category		Publications, n (%)	Citations
Document type			
	Article	12,455 (92.27)	–
	Review	1,044 (7.73)	–
Publication year			
	2015	893 (6.62)	26,701
	2016	1,167 (8.65)	31,897
	2017	1,141 (8.45)	27,645
	2018	1,295 (9.59)	27,717
	2019	1,330 (9.85)	24,670
	2020	1,413 (10.47)	21,850
	2021	1,660 (12.30)	19,089
	2022	1,541 (11.42)	11,990
	2023	1,469 (10.88)	7,439
	2024	1,590 (11.78)	2,150
Summary			
	Total	13,499 (100)	201,148
	Average	1,350	14.90
	Growth rate (2024 over 2015)	78.05%	–

### Countries/regions and institutions

**Figure 3:**
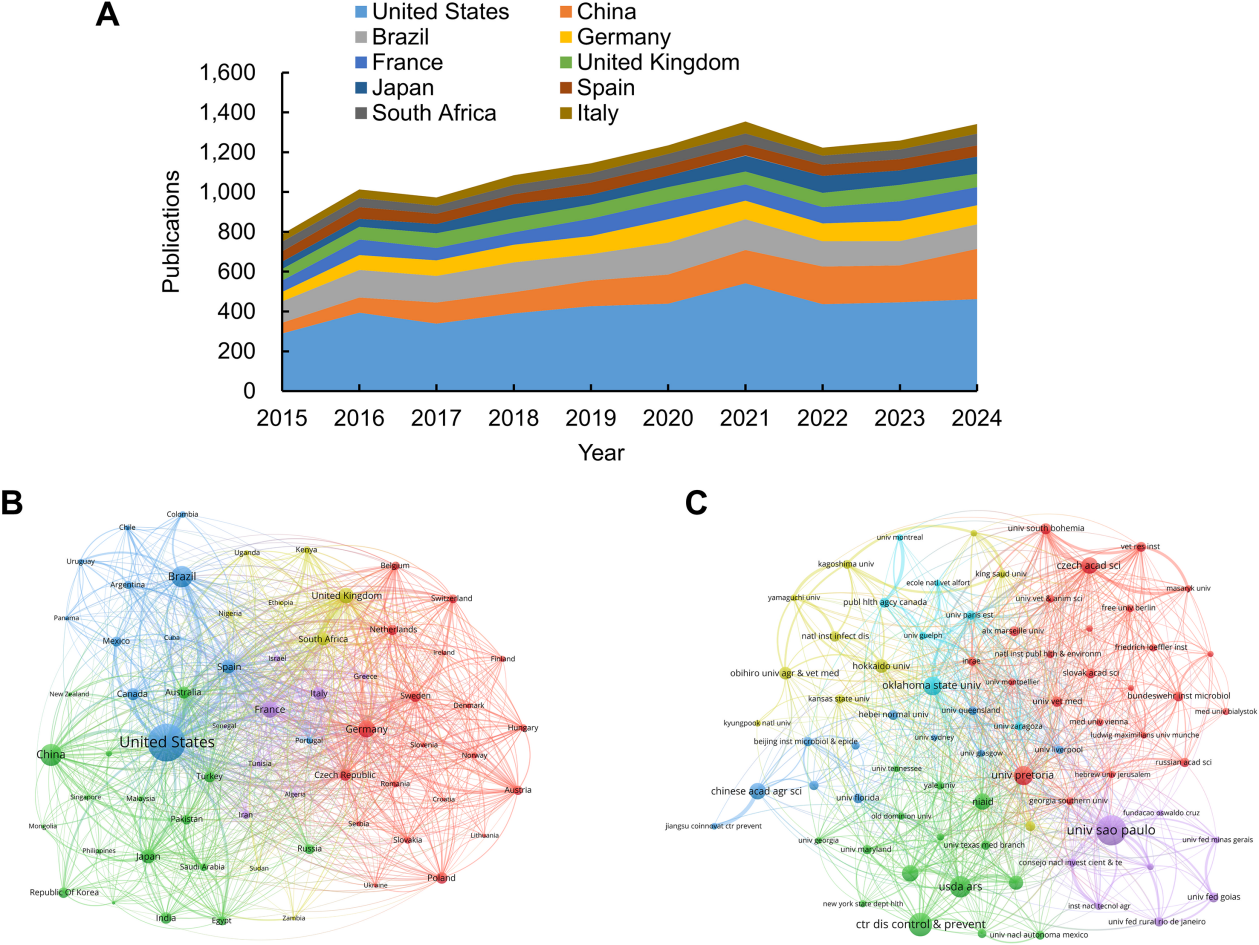
Visualization maps of countries/regions and institutions in tick research. **(A)** Trend in annual publication counts among the top 10 countries/regions from 2015 to 2024. **(B)** Co-authorship map of countries/regions involved in tick research. Node size corresponds to publication volume (range: 37–4,165); line thickness indicates collaboration strength (range: 47–2,930). **(C)** Co-authorship map of institutions involved in tick research. Node size corresponds to publication volume (range: 68–418); line thickness indicates collaboration strength (range: 12–351).

VOSviewer bibliometric analysis identified 166 countries and regions, as well as 10,850 institutions, engaged in tick-related research during the period 2015–2024. **Table 2**; **Figure 3A** list the 10 most productive countries and regions. The United States dominated the scholarly output (4,165 publications), accounting for approximately 30.85% of the total publications. China ranked second (1,412 publications), followed by Brazil (1,349), Germany (879), and France (791). The citation analysis further established U.S. leadership, with 76,814 citations, accounting for 38.62% of the total citations among the top 10 nations. To map international collaboration networks, co-authorship relationships were visualized through VOSviewer (**Figure 3B**), revealing the United States as the central hub and indicating its pivotal role in facilitating global research exchange.

**Table 2:** Top 10 largest contributing countries/regions in tick research.

Rank	Countries/regions	No.	Percentage (%)	Citations	Average citations	Link strength	H-index
1	United States	4,165	30.85	76,814	18.44	2,930	105
2	China	1,412	10.46	17,996	12.75	723	58
3	Brazil	1,349	9.99	18,262	13.54	949	53
4	Germany	879	6.51	16,896	19.22	1,314	62
5	France	791	5.86	16,148	20.41	1,280	66
6	United Kingdom	690	5.11	15,126	21.92	1,200	59
7	Japan	627	4.64	8,291	13.22	690	46
8	Spain	553	4.10	13,401	24.23	1,048	57
9	South Africa	487	3.61	7,785	15.99	747	44
10	Italy	461	3.42	8,200	17.79	619	49

The top 10 institutions that have published the largest number of papers are listed in **Table 3**. Six of the top 10 are in the United States, representing more than half of the total. Brazil, South Africa, the Czech Republic, and China each had one institution. The Universidade de São Paulo was the leading institution in terms of publications, with 418 papers, followed by the Centers for Disease Control & Prevention (CDC) in the United States, which published 315 papers, and the United States Department of Agriculture (USDA), which published 279 papers. These institutions are at the forefront of tick research, reflecting the central roles of Brazil and the United States in advancing the field. The CDC was ranked first in terms of citation performance, with 10,543 citations and an average of 33.47 citations per article. **Figure 3C** illustrates the active collaborative relationships between these institutions, highlighting their significance in this field.

**Table 3:** Top 10 most productive institutions in tick research.

Rank	Institution	Country	No.	Citations	Average citations	Link strength	H-index
1	Universidade de Sao Paulo	Brazil	418	6,927	16.57	273	41
2	Centers for Disease Control & Prevention - USA	USA	315	10,543	33.47	107	53
3	United States Department of Agriculture (USDA)	USA	279	5,107	18.30	233	39
4	University of Pretoria	South Africa	245	4,407	17.99	129	38
5	Oklahoma State University	USA	241	5,462	22.66	192	40
6	Czech Academy of Sciences	Czechia	208	4,493	21.60	351	44
7	Texas A&M University	USA	207	3,078	14.87	139	32
8	Chinese Academy of Agricultural Sciences	China	206	2,447	11.88	107	24
9	National Institute of Allergy and Infectious Diseases (NIAID)	USA	202	4,884	24.18	155	42
10	Washington State University	USA	177	2,774	15.67	162	28

### Authors and co-cited authors

**Figure 4:**
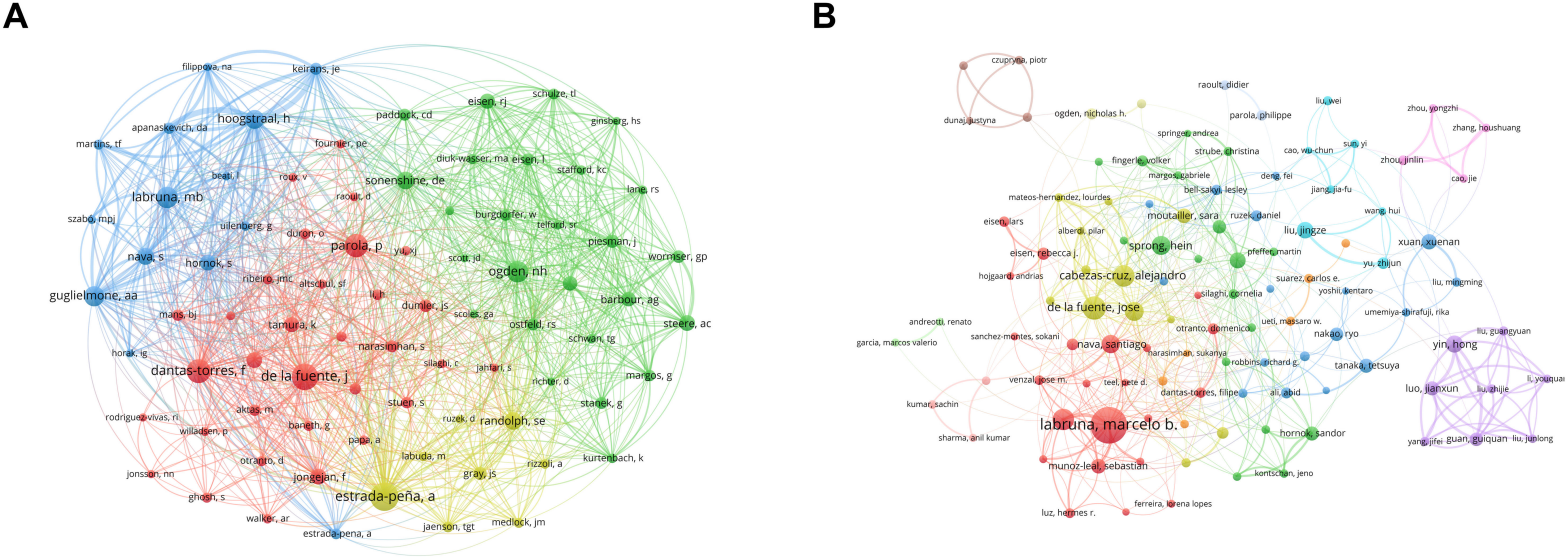
Visualization maps of co-authorship and co-citation involved in tick research. **(A)** Author co-citation network. Node size corresponds to citation frequency (range: 455–2,788); line thickness indicates co-citation strength (range: 1,102–42,128). **(B)** Author co-authorship network. Node size represents publication volume (range: 35–267); line thickness signifies collaboration intensity (range: 3–374).

According to the most recent tally, 45,027 authors have published academic papers related to research on ticks. **Table 4** lists the 10 authors who demonstrated the most prolific publication performance over the past decade. The author with the most publications is Marcelo B. Labruna (267), followed by José de la Fuente (147), Alejandro Cabezas-Cruz (136), and Thiago F. Martins (132). Notably, although Estrada-Peña from Spain ranked sixth in terms of publication count, he had the highest average citation per article at 41.44, the highest among all authors. José de la Fuente, meanwhile, ranks second in both publication volume and co-citation, indicating that his output was not only more prolific but also of higher quality and greater influence. **Figure 4** illustrates the co-authorship and co-citation network analysis of prominent authors. Authors with higher publication output are typically associated with larger collaboration networks.

**Table 4:** Top 10 most productive authors and co-cited authors in tick research.

Rank	Author	Country	No.	Total citations	Average citations	H-index	Co-cited author	Country	Citations
1	Labruna, Marcelo B.	Brazil	267	4,622	17.31	36	Estrada-Pena, A.	Spain	2,788
2	de la Fuente, Jose	Spain	147	3,926	26.71	37	de la Fuente, Jose	Spain	2,581
3	Cabezas-Cruz, Alejandro	France	136	3,097	22.77	32	Dantas-Torres, Filipe	Brazil	2,230
4	Martins, Thiago F.	Brazil	132	2,120	16.06	30	Parola, Philippe	France	2,107
5	Sprong, Hein	Netherlands	117	3,386	28.94	39	Labruna, Marcelo B.	Brazil	1,921
6	Estrada-Pena, A.	Spain	113	4,683	41.44	40	Ogden, Nicholas H.	Canada	1,891
7	Nava, Santiago	Argentina	112	1,752	15.64	23	Guglielmone, Alberto A.	Argentina	1,734
8	Yin, Hong	China	101	1,320	13.07	21	Hoogstraal, H.	USA	1,592
9	Liu, Jingze	China	97	1,133	11.68	18	Randolph, Sarah E.	UK	1,454
10	Chitimia-Dobler, Lidia	Germany	92	1,651	17.95	22	Sonenshine, Daniel E.	USA	1,428

### Journals and co-cited journals

**Figure 5:**
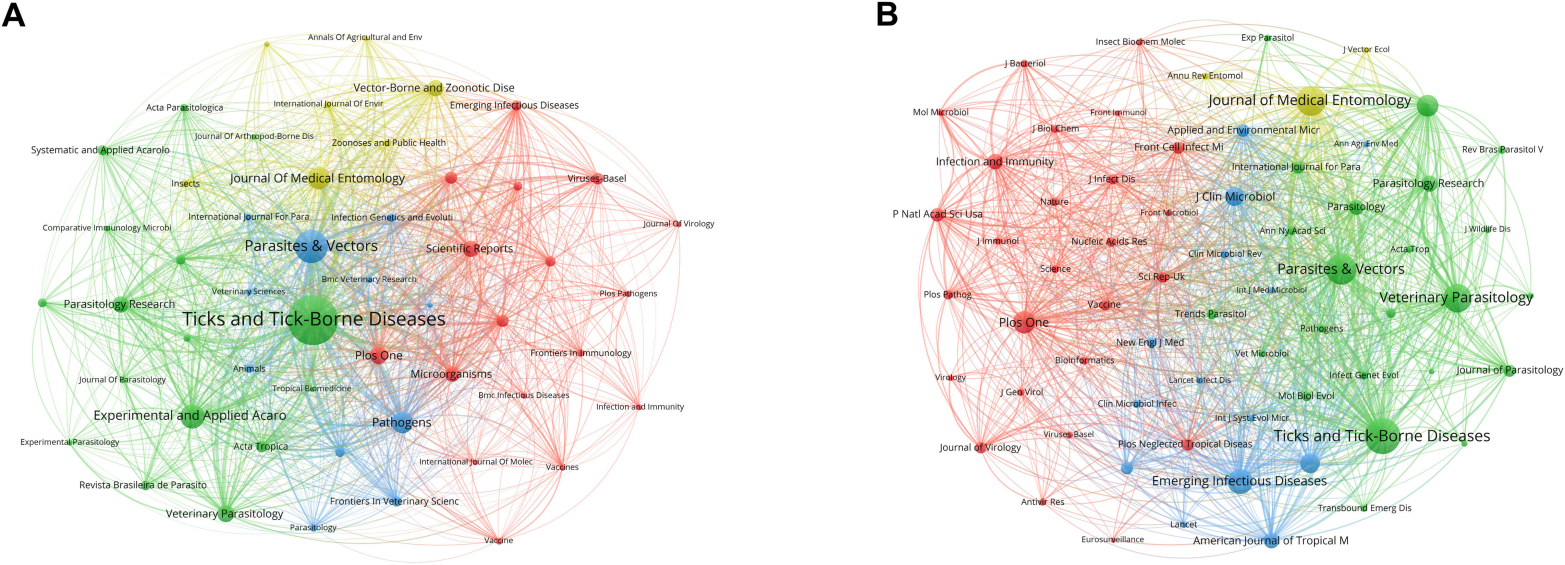
Visualization maps of relevant journals from 2015 to 2024. **(A)** Network visualization map of journals’ co-occurrence on tick research. Node size represents publication volume (range: 46–1498); line thickness indicates collaboration strength (range: 196–18,215). **(B)** Network visualization map of journal co-citation analysis on tick research. The sizes of the nodes represent the citation frequency (range: 1,543–29,376), and the lines between two nodes indicate that both were cited by the same journal.

There were 1,220 journals that published research related to tick research during the past decade. We constructed a network visualization map that showed a close collaboration between leading journals (**Figure 5A**). **Table 5** ranks the top 10 journals with the most published articles. *Ticks and Tick-Borne Diseases* is the most prolific publication, with 1,498 publications and 25,309 citations, demonstrating its central role in disseminating knowledge in this field. According to the 2023 Journal Citation Report (JCR), the majority of these journals are distributed within the Quartile 1 (Q1) or Quartile 2 (Q2) range.

**Table 5:** Top 10 journals with the most articles in tick research.

Rank	Journal	No.	Percentage (%)	Country	Citations	Average citations	Link strength	H-index	IF (2023)	JCR (2023)
1	Ticks and Tick-Borne Diseases	1,498	11.10	Germany	25,309	16.90	18,215	55	3.1	Q1/ Q2
2	Parasites & Vectors	796	5.90	UK	17,282	21.71	11,989	58	3	Q1
3	Experimental and Applied Acarology	488	3.62	Netherlands	5,577	11.43	5,527	30	1.8	Q2
4	Journal of Medical Entomology	422	3.13	USA	6,777	16.06	5,325	35	2.1	Q1
5	Pathogens	399	2.96	Switzerland	3,717	9.32	6,332	29	3.3	Q2
6	PloS One	297	2.20	USA	5,800	19.53	3,599	42	2.9	Q1
7	Veterinary Parasitology	269	1.99	Netherlands	4,561	16.96	3,057	39	2	Q2
8	Vector-Borne and Zoonotic Diseases	260	1.93	USA	3,690	14.19	2,794	33	1.8	Q3
9	Parasitology Research	255	1.89	Germany	3,325	13.04	3,101	29	1.8	Q3
10	Scientific Reports	253	1.87	UK	3,904	15.43	3,156	36	3.8	Q1

The frequency of co-citation is a significant metric for assessing the impact of a journal. The co-citation analysis showed that 66 journals were co-cited over 1500 times (**Figure 5B**). The most co-cited journal was *Ticks and Tick-Borne Diseases* (29,376 times), followed by *Parasites & Vectors* (22,554 times). The patterns of co-citation reveal a collaborative ecosystem within parasitology and acarology research, characterized by interdisciplinary research that integrates tick biology, vector control, and disease ecology.

### Cocited reference and reference bursts

**Figure 6:**
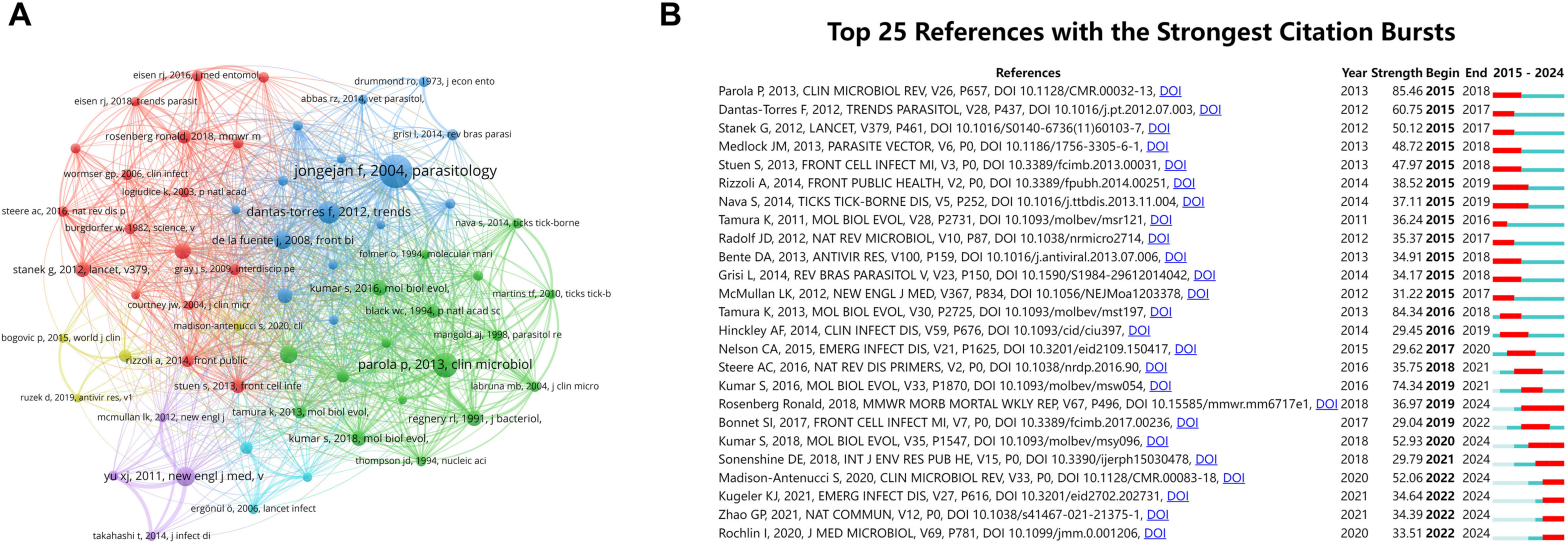
Co-citation networks and high-impact reference dynamics in tick research. **(A)** Co-citation network map of references on ticks. Node size corresponds to citation frequency (range: 202–906); line thickness indicates co-citation strength (range: 437–1,705) **(B)** Top 25 references with the highest burst activity in tick research (generated by CiteSpace). The blue bars indicate the time interval, and the red bars indicate the active time.

Co-citation analyses are employed within the field of scholarship to measure the degree of association between references within a specific research domain. VOSviewer was employed to generate a network map of the references co-cited more than 200 times (**Figure 6A**). The 10 references with the highest number of co-citations in tick research are listed in **Table 6**. The study published in *Parasitology* by Frans Jongejan in 2004 entitled “The global importance of ticks” was the most co-cited article (906 times) in this field (Jongejan and Uilenberg, 2004).

**Table 6:** Top 10 co-cited references in tick research.

Rank	First author	Title	Year	Source	Co-citations	Link strength
1	Jongejan, Frans	The global importance of ticks	2004	Parasitology	906	1,705
2	Parola, Philippe	Update on tick-borne rickettsioses around the world: a geographic approach	2013	Clinical Microbiology Reviews	651	1,598
3	Dantas-Torres, Filipe	Ticks and tick-borne diseases: a One Health perspective	2012	Trends in Parasitology	575	1,201
4	Yu, Xue-Jie	Fever with thrombocytopenia associated with a novel bunyavirus in China	2011	New England Journal of Medicine	494	871
5	de la Fuente, Jose	Overview: Ticks as vectors of pathogens that cause disease in humans and animals	2008	Frontiers in Bioscience-Landmark	457	985
6	Dumler, JS	Reorganization of genera in the families Rickettsiaceae and *Anaplasmataceae* in the order *Rickettsiales*: unification of some species of *Ehrlichia* with *Anaplasma*, *Cowdria* with *Ehrlichia* and *Ehrlichia* with *Neorickettsia*, descriptions of six new species combinations and designation of *Ehrlichia equi* and 'HGE agent' as subjective synonyms of *Ehrlichia phagocytophila*.	2001	International Journal of Systematic and Evolutionary Microbiology	434	780
7	Medlock, Jolyon M.	Driving forces for changes in geographical distribution of *Ixodes ricinus* ticks in Europe	2013	Parasites & Vectors	415	811
8	Kumar, Sudhir	MEGA7: molecular evolutionary genetics analysis version 7.0 for bigger datasets	2016	Molecular Biology and Evolution	389	594
9	Regnery RL	Genotypic identification of rickettsiae and estimation of intraspecies sequence divergence for portions of two rickettsial genes	1991	Journal of Bacteriology	383	975
10	Stanek, Gerold	Lyme borreliosis	2012	The Lancet	379	616

Using CiteSpace software, we examined the references that showed the strongest citation bursts (**Figure 6B**). Among the references with a significant surge in citations, the articles with the highest values were Parola et al. (2013); Tamura et al. (2013), and Kumar et al. (2016). The rapid evolution of Molecular Evolutionary Genetics Analysis (MEGA) underscores the need for researchers to undertake comparative analyses of DNA and protein sequences from substantial datasets. Notably, the most prominent citation surge corresponds to the second most co-cited article, with an intensity of 85.46. The majority of high-burst articles were concentrated in publications from 2012 to 2016, while more recent publications (post-2020) exhibited relatively lower burstiness, although the trend has persisted, indicating an ongoing evolution of research hotspots.

### Keywords analysis and burst research

**Figure 7:**
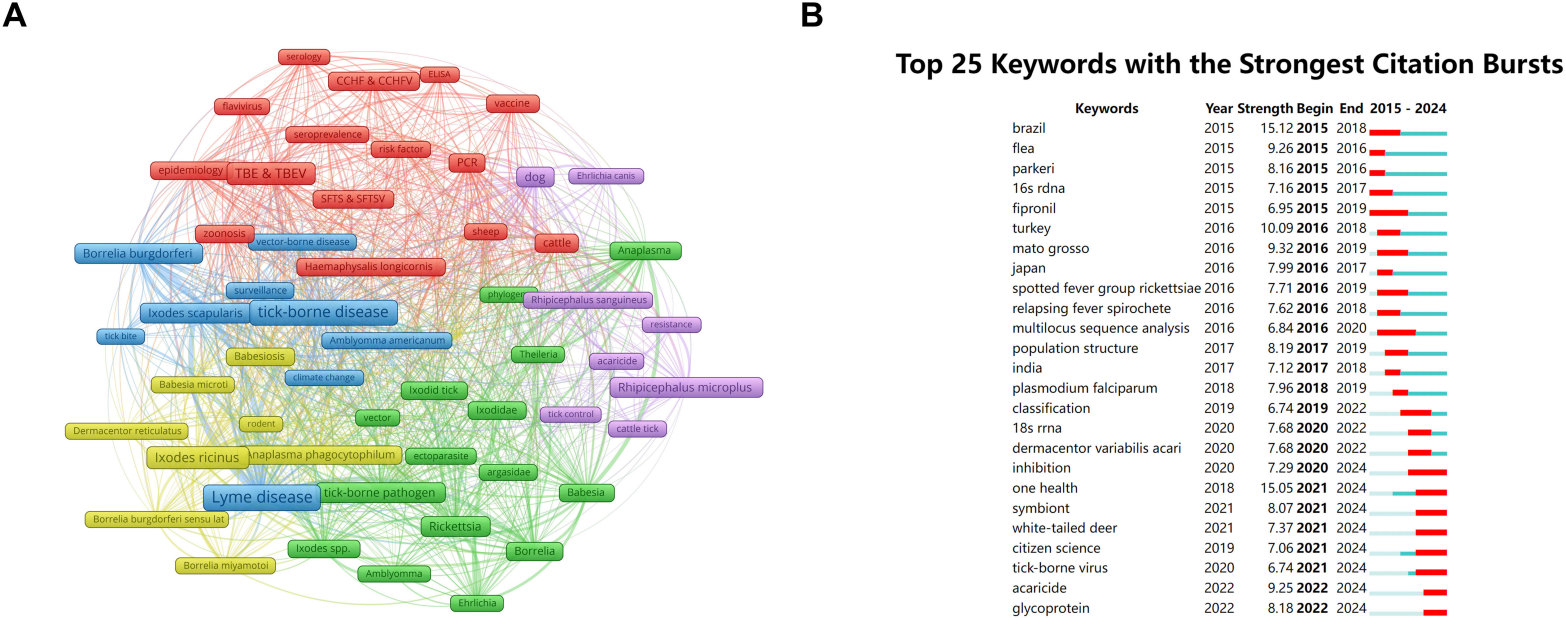
Keywords co-occurrence network and burst dynamic in tick research. **(A)** Network visualization map of keywords co-occurrence in tick research. Node size corresponds to keyword frequency (range: 104–1,062); line thickness indicates co-occurrence strength (range: 71–1,513). **(B)** Top 25 keywords with the strongest citation bursts in tick research (generated by CiteSpace). The blue bars indicate the time interval, and the red bars indicate the active time.

The keyword analysis revealed the research hotspots and scientific trends of ticks. A total of 16,977 keywords were obtained, and a subsequent analysis revealed 55 words that occurred more than 100 times (**Figure 7A**). The identified keywords could be divided into five clusters: (1) research on TBDs and pathogens, including terms such as Lyme disease, TBE, *Borrelia burgdorferi*, *Babesia*, *Rickettsia* and *Anaplasma phagocytophilum* (**Supplementary Figure S1**); (2) research on tick species and vectors, including *Ixodes ricinus*, *I. scapularis*, *Rhipicephalus microplus*, *Haemaphysalis longicornis* and *Amblyomma americanum* (**Supplementary Figure S2**); (3) research on surveillance, epidemiology, and risk factors related to TBDs, such as zoonosis and climate change; (4) research on diagnosing TBDs, including terms related to methods such as the polymerase chain reaction (PCR) and enzyme-linked immunosorbent assay (ELISA), and (5) research on vaccine development and control measures related to TBDs, including terms such as vaccine, acaricide, and resistance. **Table 7** presents a summary of the 15 most frequent keywords, with the majority of top-ranked terms involving TBDs and pathogens.

**Table 7:** Top 15 keywords involved in tick research.

Rank	Keywords	Occurrences	Link strength
1	Lyme disease	1032	1,513
2	tick-borne disease	940	1,244
3	TBE & TBEV	668	564
4	*Ixodes ricinus*	610	790
5	*Borrelia burgdorferi*	440	594
6	tick-borne pathogen	440	764
7	*Rickettsia*	414	629
8	dog	393	672
9	*Rhipicephalus microplus*	389	549
10	*Ixodes scapularis*	387	240
11	*Borrelia*	373	661
12	*Anaplasma phagocytophilum*	361	577
13	PCR	340	468
14	cattle	332	409
15	CCHF & CCHFV	297	32

TBE, tick-borne encephalitis; TBEV, tick-borne encephalitis virus; PCR, polymerase chain reaction; CCHF, Crimean-Congo hemorrhagic fever; CCHFV, Crimean-Congo hemorrhagic fever virus.

The CiteSpace software generated keywords with the strongest citation bursts (**Figure 7B**), reflecting the research frontiers and trends. The term “Brazil” exhibited the earliest citation burst, with a strength of 15.12, highlighting the geographic significance of this nation in TBDs research. Notably, while “One Health”—an interdisciplinary approach that integrates the health of humans, animals, and their shared environment—appeared in the literature as early as 2018, its citation surge peaked during 2021–2024, with a strength of 15.05, likely attributable to accelerated multidisciplinary collaborations in recent years (Zinsstag et al., 2011).

## Discussion

Studies have shown that the prevalence of TBDs and the associated risks to human and animal health have been steadily increasing (Estrada-Peña et al., 2012; Madison-Antenucci et al., 2020). Despite concerted efforts at eradication, ticks and the pathogens they disseminate persist, constituting a serious threat to human and animal health on a global scale (Jongejan and Uilenberg, 1994; de La Fuente and Kocan, 2006; Semenza et al., 2022). As the volume of related literature increases, it becomes increasingly challenging to track emerging research trends. Bibliometric analyses offer a valuable means to comprehend the growth, development, and dissemination of knowledge in tick research, with the aim of capturing emerging trends and predicting future research directions (Thompson and Walker, 2015; Kraus et al., 2024; Öztürk et al., 2024).

The present study employed a bibliometric analysis to assess global tick research, analyzing publication data to identify research hotspots and trends. A comprehensive analysis of 13,499 articles published over the past decade revealed a significant upward trend in the number of publications, suggesting a growing focus on tick research. However, this growth has not been mirrored by an increase in the number of citations. The disparity between publications and citations suggests a delay in the recognition of newer research; this is expected as the academic community processes and integrates findings over time. The top five countries accounted for more than 60% of the publications. The United States has assumed a prominent position in tick research, exhibiting a higher volume of publications compared to other nations. This may be attributed to the extensive history of tick research in the United States and the high prevalence of TBDs (Beard et al., 2019; Kugeler et al., 2021), which is exemplified by the seminal article on *Babesia bigemina* published by Smith and Kilborne in 1893 (Smith and Kilborne, 1893). China has exhibited the most rapid growth in tick research, which can be attributed to the rising frequency of new TBDs and the growing burden of various existing tick-borne illnesses (Wu et al., 2025). Marcelo B. Labruna from the University of São Paulo ranked first in the number of publications, and Estrada-Pena from the University of Zaragoza ranked first among co-cited authors.

Over the past decade, research on TBDs and pathogens has remained at the forefront of the field. Important TBDs, such as Lyme disease (caused by *B. burgdorferi*), TBE (caused by TBEV), Rocky Mountain spotted fever (caused by spotted fever group rickettsiae), and babesiosis (caused by protozoan parasites within the genus *Babesia* spp.), have been extensively studied. Numerous epidemiological surveys and molecular studies have revealed the transmission routes, pathogenesis, and genetic diversity of pathogens responsible for these classic TBDs. At the same time, global warming has led to a significant expansion of the geographic distribution of ticks, while the fragmentation of wildlife habitats and the acceleration of international trade and tourism have led to the emergence of new TBDs. Human granulocytic anaplasmosis (HGA) is a well-documented tick-borne zoonosis. The initial report occurred in the United States in 1990, and the disease was confirmed in Europe in 1997 (Zhang et al., 2008; Nepveu-Traversy et al., 2024). The geographic distribution of the causative agent, the phagocytic *A. phagocytophilum*, and its primary vector, the castor tick, has been expanding, encompassing nearly the entirety of continental Europe and the Atlantic Ocean (Stuen et al., 2013). Crimean-Congo hemorrhagic fever virus (CCHFV) was originally endemic to Africa, Asia, the Middle East, and Southeastern Europe; in recent years, the emergence of cases of indigenous transmission in European countries such as Spain suggests that the disease has spread to temperate regions (Shahhosseini et al., 2021). Severe fever with thrombocytopenia syndrome (SFTS) is caused by a novel Bandavirus (Dabie bandavirus), and it is now endemic in many East Asian countries since it was first reported in China in 2009 (Yu et al., 2011; Kim et al., 2024). However, the virus’s complete life cycle in nature, its animal host spectrum, and its tick-mediated transmission mechanism have not been fully elucidated, and there is no commercial vaccine or standardized treatment regimen for the virus (Nepveu-Traversy et al., 2024). In addition, other pathogens have emerged, such as Kyasanur forest disease virus (the case range increased dramatically in 2005), Heartland virus (discovered in Missouri, USA in 2009), Jingmen tick virus (discovered in China in 2010), Alongshan virus (first described in Northeast China in 2017), and others (Qin et al., 2014; Bosco-Lauth et al., 2015; Gurav et al., 2018; Wang et al., 2019). The geographical range of classic tick-borne pathogens is expanding, and new pathogens are emerging. These factors have contributed to the complexity of public health prevention and control, presenting novel challenges related to early detection, molecular typing, and specific treatment.

Concurrently, advancements in pathogen detection methodologies have facilitated substantial progress in tick research. Initially, conventional methods such as microscopic examination, culture isolation, and serological identification were utilized (Tokarz and Lipkin, 2021). However, these techniques have limitations in detecting low-abundance or novel pathogens. The advent of PCR technology has enabled researchers to screen tick samples for specific pathogens in a more cost-effective, time-efficient, and productive manner (Telford et al., 1997; Sparagano et al., 1999). This was followed by the advent of next-generation sequencing (NGS), which enabled the detection of pathogens to be expanded beyond the confines of a single target. This development facilitated macro-genomics analyses, leading to the concurrent identification of multiple pathogens and their co-existence in larger samples (Carpi et al., 2011; Andreotti et al., 2011; Trout Fryxell and DeBruyn, 2016). This technological innovation has precipitated a shift in tick research from single-target detection to a large-scale, multi-target surveillance model. This has led to significant improvement in the ability to understand disease transmission trends and the feasibility of developing early warning systems. Since then, a new trend in TBDs research has emerged, thanks to the development of advanced genomic tools, including NGS. Researchers have gained a deeper level of comprehension regarding the tick microbiome and endosymbionts within ticks, as well as their functions in tick biology, including immune regulation and the transmission of pathogens (Narasimhan and Fikrig, 2015; Gurfield et al., 2017). These endophytic bacteria have important implications for tick development, survival, and ability as pathogen vectors (Hussain et al., 2022; Kolo and Raghavan, 2023). Through deeper excavation of the tick microbiome interaction network, researchers have been able to analyze the complex linkages between ticks, symbiotic bacteria, and pathogens and provide a basis for future strategies based on intervening with symbiotic bacteria to block pathogen transmission.

The analysis of keywords revealed several noteworthy patterns and emerging hotspots in the field. Between 2015 and 2018, keywords such as “Brazil” (burst intensity: 15.12) and “Turkey” (10.09) experienced frequent bursts, reflecting a geographical shift in research focus. Brazil’s prominent position is closely linked to ecological disturbances caused by the development of the rainforest. Tropical rainforest fragmentation has forced the migration of tick hosts to human-populated areas and the consequent spread of tick-borne pathogens such as *Rickettsia rickettsii* to human settlements, thereby increasing the risk of human exposure to ticks and the diseases they transmit (Szabó et al., 2009; Dantas-Torres et al., 2019; Fonseca et al., 2020; Fornazari et al., 2021). This trend was further exacerbated by the 2015 Zika virus outbreak in May 2015 (Campos et al., 2015), and the ensuing global public health crisis spurred international collaboration in vector-borne disease research (including TBDs), positioning South America as a new hotspot for emerging research. As tick research expands on a global scale, patterns of collaboration across geographic and disciplinary boundaries are becoming clear. Although the concept of “One Health” was introduced in the literature many years ago (Nzietchueng et al., 2023), its burst period was delayed until 2021–2024 (burst intensity 15.05), likely related to the frequent occurrence of new TBDs worldwide. The global pandemic exposed the vulnerability of the human–animal–environment interface and accelerated the development of interdisciplinary collaborative mechanisms (Sprong et al., 2018). This has led to a growing recognition that pathogen ecology and disease management require an integrated approach. It is imperative to expand the scope of research from ticks and the pathogens they carry to encompass the complex interactions between humans, animals, and the ecosystem. This necessitates policy adjustments and technological innovations on a global scale (Kading et al., 2018). This transition marks a pivotal advancement in the domain of tick research, with a shift from conventional disease surveillance toward a more integrated and systematic approach. This trend has laid the groundwork for the development of a collaborative control system that incorporates human medicine, veterinary science, and environmental science.

In recent years, the keyword “acaricides” (burst intensity 9.25) has garnered increasing attention, indicating the urgency of addressing the global crisis in disease resistance. In recent decades, the widespread and intensive use of various acaricides has led to the development of resistance in many species (Wyk et al., 2016; Rodriguez-Vivas et al., 2018; Dzemo et al., 2022; De Rouck et al., 2023). Moreover, increasing evidence suggests that strategies heavily reliant on acaricides are not cost-effective, and that these chemicals have detrimental environmental impacts, including residual chemical residues in livestock products (Graf et al., 2004; Jeschke, 2021). This clearly contradicts the “One Health” concept. These issues highlight the limitations of chemical pest control and have motivated researchers to seek alternatives to conventional acaricides, accelerating the implementation of new technologies to address pesticide resistance. Host immunity has emerged as a promising alternative. In years past, researchers verified the potential of anti-tick vaccines in diminishing tick populations and curtailing the transmission of certain diseases (Canales et al., 1997; de La Fuente and Kocan, 2006). In recent years, a substantial body of research has emerged that attests to the effectiveness of various vaccines (de La Fuente and Ghosh, 2024; Nepveu-Traversy et al., 2024). However, challenges in tick vaccinology remain.

The issues associated with climate change, including rising surface temperatures and greenhouse gas emissions, are becoming increasingly severe. As is the case with numerous other arthropods, ticks are sensitive to climatic change. Studies have suggested that higher temperatures may cause shifts in the geographical range of certain tick species (Lindgren and Gustafson, 2001; Karbowiak, 2014; Clow et al., 2017). The expansion of tick habitats has resulted in an escalation in the prevalence of numerous TBDs (Lukan et al., 2010; Monaghan et al., 2015; Dahlgren et al., 2016). As a result, there is growing attention being given to tick control and vector management strategies, especially in the context of global public health crises. Encouragingly, citizen science has emerged as an efficient tool for generating large-scale datasets regarding tick distribution, complementing data collection efforts by researchers (Eisen and Eisen, 2021; Ballman et al., 2023).

The data presented herein were derived from the WoSCC and Scopus databases, thereby ensuring the accuracy and authenticity of the search results. However, despite these strengths, our study has certain limitations. First, although these databases are extensive, they may not encompass all relevant journals, particularly those with a regional focus or outside the mainstream academic sphere. Consequently, some pertinent research may have been overlooked. Second, our analysis is limited to articles and reviews, excluding other valuable sources such as conference proceedings or books, which may offer additional insights. Furthermore, excluding non-English articles may introduce language and regional biases, thereby limiting the representativeness of research from non-English-speaking regions. Finally, similar to any classical bibliometric analysis, our study is subject to the challenges of subjective bias during manual analysis, as well as potential errors in data cleaning and processing.

## Conclusion

The publication of scholarly articles on tick research has exhibited a gradual upward trend over the past decade, and TBDs and their associated pathogens remain at the center of the research landscape. Global climate change has reshaped the tick’s ecological niche, manifested as an expanding geographic distribution and significantly longer active seasons. This has directly led to the emergence of new TBDs and pathogens worldwide, creating challenges in the diagnosis and treatment. The accelerated development of molecular diagnostic techniques has facilitated the identification of novel pathogens, a development that may also underlie the rising number of reports concerning new pathogens. At the same time, traditional tick control strategies are facing serious challenges. The sustainability of chemical insecticides is being questioned, and the development of vaccines against ticks or pathogens is rapidly emerging as a promising alternative control option due to the potential specificity and environmental benefits of vaccines. In conclusion, these visualized data reveal the evolution of tick research, transitioning from a macroscopic to a microscopic perspective, from singular to multifaceted approaches, and from local to integrative frameworks. In the future, tick research will continue to evolve, becoming more interdisciplinarily, with increasing collaboration between various fields, including environmental science, molecular biology, and public health. Subsequent research efforts will prioritize genetic studies, innovative vector control methodologies, and the One Health framework to mitigate the international burden of TBDs.

## Data Availability

The original contributions presented in the study are included in the article/**Supplementary Material**. Further inquiries can be directed to the corresponding authors.

## Supplementary material

Supplementary Figure 1 Top 20 tick-borne diseases **(A)** and tick-borne pathogens **(B)** in the relevant research literature.

Supplementary Figure 2 Top 20 tick species in terms of number of relevant research literature.
