# Supplementary figures and images for: Global trends in tick research: a comprehensive visualization and bibliometric study (2015–2024)
Source: Front Cell Infect Microbiol. 2025 Oct 28;15:1697791. doi: 10.3389/fcimb.2025.1697791 (PMC12602484; doi:10.3389/fcimb.2025.1697791)

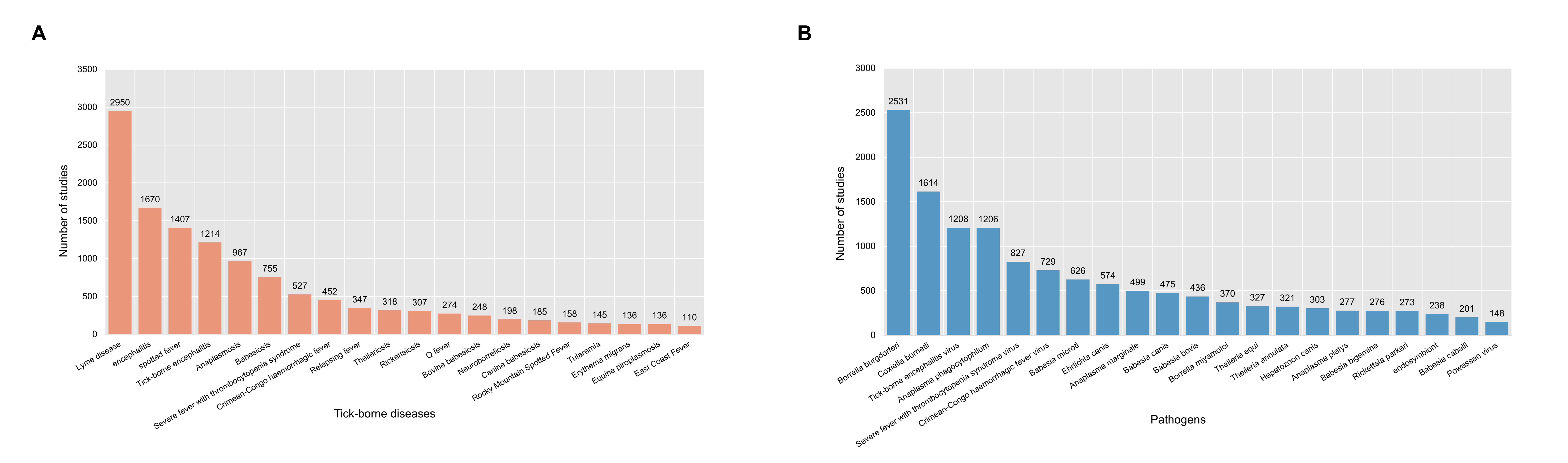

Supplement: Supplementary Figure 1 — Top 20 tick-borne diseases (A) and tick-borne pathogens (B) in the relevant research literature. [file Image1.tif]

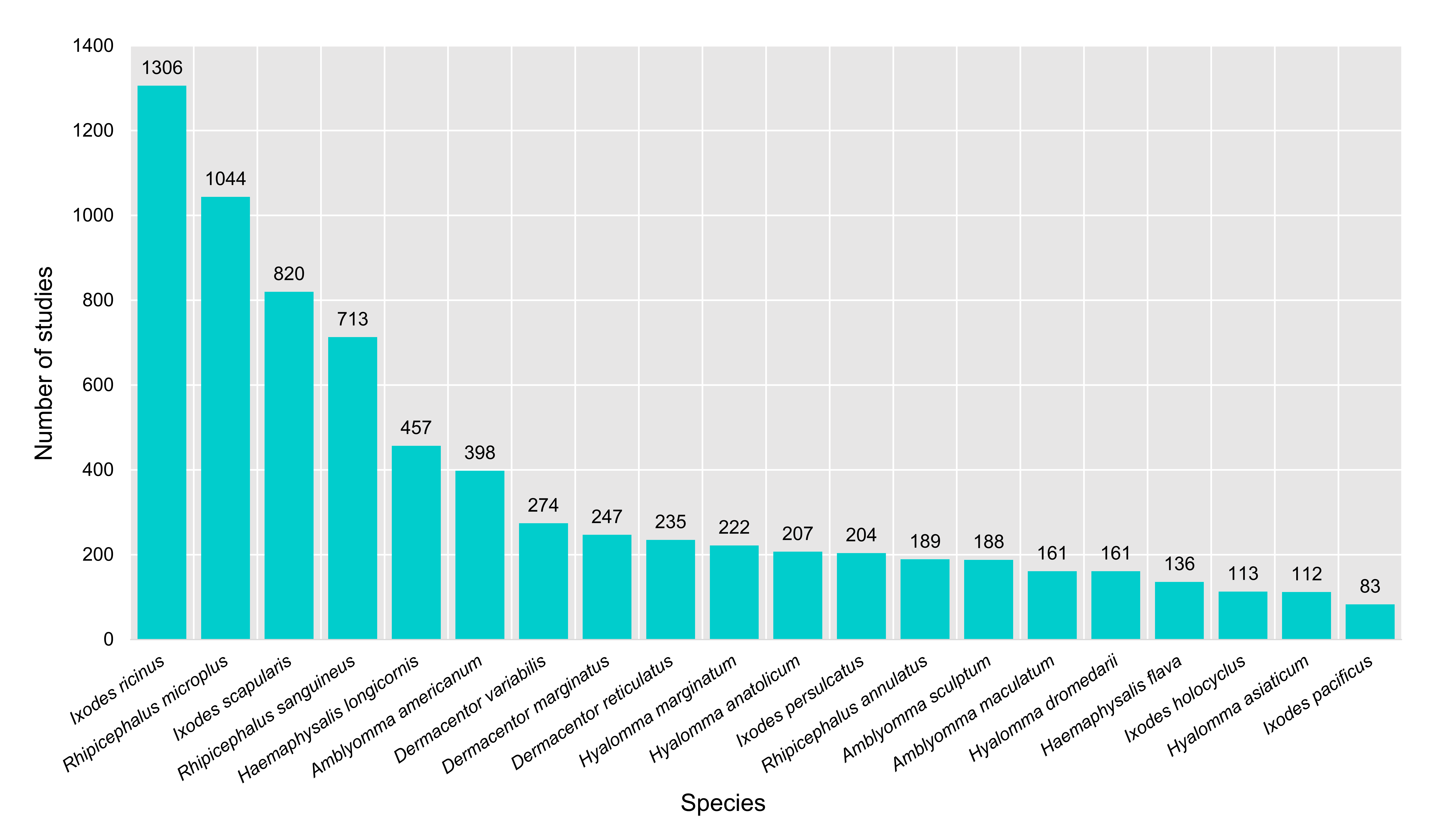

Supplement: Supplementary Figure 2 — Top 20 tick species in terms of number of relevant research literature. [file Image2.tif]
